# Genetic structure and population diversity of *Phytophthora infestans* strains in Pacific western Canada

**DOI:** 10.1007/s00253-024-13040-6

**Published:** 2024-02-26

**Authors:** Segun Babarinde, Rishi R. Burlakoti, Rick D. Peters, Khalil Al-Mughrabi, Amy Novinscak, Sanjib Sapkota, Balakrishnan Prithiviraj

**Affiliations:** 1 Agassiz Research and Development Centre, Agriculture and Agri-Food Canada, 6947 Hwy 7, Agassiz, BC V0M 1A0 Canada; 2 Department of Plant, Food and Environmental Sciences, Faculty of Agriculture, Dalhousie University, Truro, NS B2N 5E3 Canada; 3 Agriculture and Agri-Food Canada, 440 University Avenue, Charlottetown, PE C1A 4N6 Canada; 4 New Brunswick Department of Agriculture, Aquaculture and Fisheries, 39 Barker Lane, Wicklow, NB E7L 3S4 Canada

**Keywords:** Late blight, Genetic diversity, *Phytophthora infestans*, Sexual recombination

## Abstract

**Abstract:**

Late blight caused by *Phytophthora infestans* is an economically important disease of potato and tomato worldwide. In Canada, an increase in late blight incidence and severity coincided with changes in genetic composition of *P. infestans*. We monitored late blight incidence on tomato and potato in Pacific western and eastern Canada between 2019 and 2022, identified genotypes of *P*. *infestans*, and examined their population genetic diversity. We identified four major existing genotypes US11, US17, US8, and US23 as well as 25 new genotypes. The US11 genotype was dominant in Pacific western Canada, accounting for 59% of the total population. We discovered the US17 genotype for the first time in Canada. We revealed a higher incidence of late blight and quite diverse genotypes of *P*. *infestans* in Pacific western Canada than in eastern Canada. We found high genetic diversity of *P. infestans* population from Pacific western Canada, as evidenced by the high number of multilocus genotypes, high values of genetic diversity indices, and emergence of 25 new genotypes. Considering the number of disease incidence, the detection of diverse known genotypes, the emergence of novel genotypes, and the high number of isolates resistant to metalaxyl-m (95%) from Pacific western Canada, the region could play a role in establishing sexual recombination and diverse populations, which could ultimately pose challenges for late blight management. Therefore, continuous monitoring of *P. infestans* populations in Pacific western region and across Canada is warranted.

**Key points:**

*• Genotypes of P. infestans in Pacific western were quite diverse than in eastern Canada.*

*• We* *discovered US17 genotype for the first time in Canada and identified 26 novel genotypes.*

*• Approximately 95% of P. infestans isolates were resistant to metalaxyl-m.*

**Supplementary Information:**

The online version contains supplementary material available at 10.1007/s00253-024-13040-6.

## Introduction 

Potato is a valuable crop as a staple food source and for the production of textiles, pharmaceuticals, and therapeutics (Llorente et al. 2010). Potato is produced in all Canadian provinces with 40, 38, and 22% in area of potato production in the Prairie provinces and British Columbia (BC), Atlantic Canada, and Central Canada, respectively (Anonymous 2021a). Potato contributed $1.4 billion in farm cash receipts in Canada and $2 billion in exports of potato and potato products in 2020 (Anonymous 2021a). Tomato is also grown as an annual crop in most Canadian provinces, with the highest production in Ontario (ON), followed by BC and Quebec (QC) in 2020, and the summer months are the peak production periods (Anonymous 2021b). In Canada, tomato production ranks first among greenhouse vegetables and contributes about $665.9 million in farm gate values (Anonymous 2021b).

Several diseases cause adverse impacts on the yield and quality of potato and tomato in Canada and worldwide (Soylu et al. 2006; Birch et al. 2012; Arafa et al. 2022). Of these, late blight caused by *Phytophthora infestans* (Mont.) de Bary is the most destructive and economically important disease of potato and tomato. It can destroy an entire field within a week when severe epidemics occur favored by low to moderate temperature (15 to 22 °C), high relative humidity (> 90% RH), and rainfall (Haverkort et al. 2008; Fry 2020; Hansen et al. 2016a; Arafa et al. 2022), resulting in global losses up to $6.7 billion annually. The pathogen caused the Great Famine in Ireland in the nineteenth century, which resulted in the death of over a million people and the mass emigration of more than a million (Fry et al. 1993; Rekad et al. 2017). In Canada, late blight of potato and tomato is widespread and usually occurs yearly with the highest pressure in eastern provinces, while it is generally localized with low to moderate pressure in BC and Alberta (AB) (Anonymous 2021a, b). Millions of dollars have been spent on fungicide applications worldwide to protect potato and tomato from late blight (Haverkort et al. 2008; Fry et al. 2013). Studies from Finland showed that the number of fungicide sprays to manage late blight in potato increased eight-fold from 1983 (0.5 sprays) to 2002 (4 sprays) in one season (Hannukkala et al. 2007).

*P.* *infestans* has both asexual and sexual life stages. The pathogen is a heterothallic oomycete with two designated mating types A1 and A2. Sexual populations with diverse genetic diversity were reported from the highlands of central Mexico and northwest Europe (Grunwald and Flier 2005; Widmark et al. 2011). In contrast, asexual populations dominate in Canada, the USA, and the majority of other potato and tomato-growing areas worldwide (Fry 2016). Despite the predominance of asexual population, high genetic diversity was reported in North America (Kalischuk et al. 2012; Peters et al. 2014; Saville and Ristaino 2019; Saville et al. 2021), which could be due to mutation, mitotic recombination, and gene conversion (Abu-El Samen et al. 2003; Maurice et al. 2019). It is speculated that sexual recombination contributes little to disease epidemics in most of the regions of North America, though both mating types were found occasionally in a few locations (Fry 2016). For example, both mating types were found in ON in 2011 and BC in 2012 and 2013, indicating the potential of evolving sexual strains of *P*. *infestans* in Canada (Peters et al. 2014). However, further studies are required for verification. Nevertheless, late blight management will be difficult and complex if oospores exist in a field because the spores are hard and thick-walled and can tolerate adverse environmental conditions and survive several years in the soil (Mayton et al. 2000).

Several markers including allozymes of glucose-phosphate isomerase (Gpi), mating type, restriction fragment length polymorphism (RFLP) RG57 fingerprinting, and simple sequence repeats (SSR) or microsatellites have been used for characterization of *P. infestans* (Peters et al. 2014; Alkher et al. 2015)*.* In North America, strains (genotypes) of *P*. *infestans* were traditionally identified using allozyme banding patterns and RFLP. RFLP fingerprinting is laborious, time-consuming, and technically difficult. Therefore, SSR markers have been preferred for genotyping *P*. *infestans* in recent years, as multiplexing of all pairs of SSR primers can be done in a single reaction, allowing genotyping in a short period of time (Saville and Ristaino 2019). In addition, SSR markers are highly polymorphic, affordable, reproducible, neutral, co-dominant, and easy to automate and score (Lees et al. 2006; Li et al. 2013; Arafa et al. 2018). Furthermore, SSR genotyping can identify the pathogen genotypes directly from infected tissues without isolating the pathogen (Guichoux et al. 2011; Li et al. 2013).

In Canada, displacement of previous populations of *P*. *infestans* by new populations was reported in 1994–1996 (Punja et al. 1998) and 2009–2011 (Peters et al. 2014). In 1994–1996, the mefenoxam-sensitive strain US1 was displaced by the mefenoxam-resistant strain US8. In 2009–2011, US8 strains were displaced by the US23 strains. New strains including US22 and US24 were also reported first in 2010 and later in 2011 (Alkher et al. 2015; Peters et al. 2014). Diverse strains including US7, BC11, BC13, BC14, and BC15 were found between 1993 and 1997 in BC (Punja et al. 1998); after that, no comprehensive studies were conducted to understand the population dynamics of* P*. *infestans* strains. From several regions of the world, it is reported that changes in strains or genotypes of *P. infestans* and their migration to new areas or regions lead to increased incidence and outbreaks of late blight of potato and tomato. For example, the 2009 pandemic of late blight in the northeast USA occurred due to the clonal lineage US22, and the 2014 late blight outbreak in West Bengal, India, occurred due to strain 13_A2 (Fry 2016; Saville et al. 2016). These epidemics underscore the importance of continuing the study of *P*. *infestans* populations to understand population dynamics and tailor late blight management programs and strategies. Therefore, the research objectives of this study were to (i) assess late blight occurrence in potato and tomato over multi-years from diverse regions in Pacific western Canada, (ii) identify the genotypes of *P*. *infestans* populations in Pacific western and eastern provinces of Canada, and iii) understand the genetic diversity and population structure of *P. infestans* isolates from the Pacific western Canada.

## Materials and methods

### Disease monitoring, sampling, and pathogen isolation and identification

Tomato and potato commercial fields, small farms, field plots in research centers, and community gardens from diverse locations in BC were visited during June to November between 2019 and 2021. Percent severity of late blight was recorded from the spots or plots where the disease was observed and plant tissue samples showing late blight symptoms were collected. Pathogen-infected samples collected from commercial potato fields included the potato varieties Kennebeck, AC Peregrine, Gemstar Russet, Russet Norkotah, and Warba. Potato samples were also collected from small farms, research centers, and a few community gardens, whereas most of the tomato samples were from community gardens and a few from small farms and home gardens. Potato leaf and stem samples and tomato leaf, stem, and fruit samples showing symptoms of late blight were collected from each field. The samples were packed in Ziploc plastic bags and stored at 4 °C before processing for pathogen isolation. Sampling details including numbers of samples, fields, host, locations, and years of collections are summarized in Table 1.

**Table 1:** Details of sampling locations, hosts, and years of collection of *Phytophthora infestans* isolates and frequency of their genotypes

Location^1^	Year	Host	Field type^2^	No. of fields	No. of samples	No. of isolates	No. of isolates for each genotype^3^				
							US8	US 11	US 17	New	(New genotype name)
Abbotsford	2019	Tomato	CGs	2	9	22		8		14	3 (CAC6), 10 (CAC4), 1 (CAC5)
	2019	Potato	CGs	1	1	2				2	2 (CAC6)
	2020	Tomato	CGs	1	4	11		11			
	2020	Potato	CGs	1	1	3	3				
	2021	Tomato	CGs	1	3	15		12	3		
Subtotal				6	18	53	3 [6%]	31 [58%]	3 [6%]	16 [30%]	
Agassiz	2019	Potato	RF	2	3	10	1	5	3	1	1 (CAC11)
	2019	Tomato	CGs	1	1	10		6		4	1 (CAC2), 1 (CAC8), 1 (CAC9), 1 (CAC11)
	2021	Tomato	CGs	1	5	12		3	2	7	5 (CAC24), 1 (CAC25), 1 (J12)
Subtotal				4	9	32	1 [3%]	14 [44%]	5 [16%]	12 [38%]	
Chilliwack	2019	Tomato	CGs, HGs	4	5	14		12	1	1	1 (CAC3)
	2019	Potato	CFs	1	1	2		2			
	2020	Tomato	CGs, HGs	3	5	14		14			
	2020	Potato	CGs	1	1	1		1			
Subtotal				9	12	31		29 [94%]	1 [3%]	1 [3%]	
Cloverdale	2019	Potato	CFs	1	1	10		7 [70%]	2 [20%]	1 [10%]	1 (CAC12)
Delta	2020	Potato	CFs	3	5	20	3 [15%]	3 [15%]	3 [15%]	11 [55%]	10 (CAC13), 1 (CAC19)
Pitt meadow	2019	Potato	CGs	1	1	4		1		3	1 (CAC1), 1 (CAC5), 1 (CAC10)
	2019	Tomato	CGs	1	2	9		7		2	1 (CAC2), 1 (CAC7)
	2020	Tomato	CGs	1	8	32		22	1	9	4 (CAC14), 2 (CAC16), 1 (CAC17), 1 (CAC18), 1 (CAC24),
Subtotal				3	11	45		30 [67%]	1 [2%]	14 [31%]	
Richmond	2020	Potato	CFs	1	1	7		1 [14%]		6 [86%]	1 (CAC15), 1 (CAC20), 1 (CAC21), 2 (CAC22), 1 (CAC23),
Vancouver Island	2020	Tomato	HGs	1	4	6		6 [100%]			
Total				28	61	204	7 [3%]	121 [59%]	15 [7%]	61 [30%]	

^1^All locations were from British Columbia, Canada; ^2^*CGs* community gardens, *HGs* home gardens, *CFs* commercial farms, *RF* research farm; ^3^genotypes were identified from simple sequence repeat (SSR) genotyping, genotype names initiated with letter “CAC” are new genotypes

To isolate the pathogen, foliar tissue samples (leaves, stems) of potato and tomato, as well as fruits of tomato showing late blight symptoms, were washed with running tap water, and pieces (~ 3 cm × 4 cm) of the plant tissues with healthy and infected portions were cut and surface sterilized using 75% ethanol for 40 s, then immersed in 10% commercial bleach for 45 s, and finally rinsed three times with sterile water. These sterile plant tissues were kept in a moist chamber (Petri dishes with moist filter paper) and incubated at 18 °C for 3–5 days. Incubated plant samples were checked under a binocular microscope. Small pieces of mycelium with typical lemon-shaped sporangia were transferred onto either V8 rye PAR (Sapkota et al. 2023) or pea agar (120 g of frozen peas, 15 g of agar, 1 L of distilled water amended with 200 $\mu$ l pimaricin, 250 mg ampicillin, 400 $\mu$ l rifampicin). Plates were incubated for 2–4 weeks, and colony growth was examined and further purified as needed. Each isolate was obtained from individual lesions of infected plant samples, and the hyphal tip method was used to obtain each isolate after purification. Pathogen isolates were identified as *P. infestans* using colony morphology on V8 rye PAR and pea agar (white coenocytic non-septate mycelia), as well as sporangia morphology (oval-shaped, ellipsoidal to lemon-shaped, spindle-like in the base and semi-papillate) (Gallegly and Hong 2008). Reference isolates of *P*. *infestans* from previous studies (Peters et al. 2014) were also included as positive controls for identifying unknown isolates. All isolates recovered in this study were maintained on half-strength acidified V8 rye PAR or pea agar for short-term use. For long-term storage, isolates were grown on rye seeds placed on V8 rye PAR, and infected rye seeds were then placed in 2-mL vials with sterile water and stored at 10 °C. All the strains (Strain IDs: CAPi1- CAPi214) used in this study were deposited in the long-term storage of Plant Pathology Laboratory in Agassiz and Charlottetown Research Centres of Agriculture and Agri-Food Canada, which are public research institutes.

### Weather data

Daily temperature and rainfall were recovered using weather stations at the Agassiz Research and Development Centre (ARDC) in Agassiz and substations at Abbotsford, Pitt Meadows and Vancouver, BC. The weather stations were equipped with temperature and relative humidity sensors (SEN-R Combisensor Temp/rh Adcon TR1) and rain gauges (Adcon Telemetry, Klosterneuburg, Austria) to log weather data in near real time at 15-min interval. Average rainfall and temperature data were calculated using daily data and weather stations that were in operation between 2019 and 2021.

### Pathogen DNA extraction

Agar plugs of individual *P. infestans* isolates were taken from the edge of actively growing colonies on pea agar and transferred to pea broth supplemented with antibiotics (120 g of frozen peas, 1 L of distilled water amended with 200 $\mu$ l pimaricin, 250 mg ampicillin, 400 $\mu$ l rifampicin) in (15 mm × 15 mm) Petri plates. The Petri plates were incubated in a growth chamber at 16 °C for 2 to 3 weeks. Mycelia were harvested by filtering the broth through a Buchner funnel containing filter paper (Whatman no. 1, Kent, UK). Mycelia of isolates were freeze-dried overnight or for 2 days and stored at − 20 °C until further use. Genomic DNA was extracted using freeze-dried mycelia of each isolate as described by Goodwin et al. (1992) and Fry (2013). The quantity of the extracted DNA samples was measured using Qubit (Invitrogen, CA, USA).

### Preparation of infected plant tissue for genotyping

Using Whatman’s FTA (Flinders Technology Associates) cards, late blight-infected potato tissues were prepared for SSR genotyping of *P.* *infestans.* This technique does not require culturing the pathogen, and infected plant tissue can be used directly. We processed six samples on FTA card for SSR genotyping in this study to determine the reliability of the method, as SSR genotyping using FTA card directly from infected late blight samples was not commonly used in Canada. To process the sample, the margins of actively spreading lesions of infected plant tissues were pressed onto FTA cards (Whatman Inc., Kent, UK) by applying moderate rubbing pressure using a pestle. The processed FTA cards were air-dried and stored in a desiccator until further use. Samples from FTA cards were processed and cleaned as described by Burlakoti et al. (2020). Briefly, two pieces of FTA disc (2 mm diameter size) were punched using a Harris Uni-Core™ (Ted Pella Inc., CA, USA), then were kept in a 1.5-ml microcentrifuge tube, and purified according to the manufacturer’s instructions (Whatman® FTA® Card Technology, Kent, UK). Purified pieces of card were placed directly as DNA templates into the PCR mix for the multiplex SSR genotyping.

### Multiplexed SSR genotyping

Multiplex SSR genotyping of *P. infestans* isolates (*n* = 210) and pathogen-infected potato samples from Delta obtained in 2022 processed on FTA cards (*n* = 6) from this study and four reference isolates, US8, US11, US23, and US24 from Peters et al. (2014), were amplified using 12 sets of multiplex SSR primers as described in Li et al. (2013) and Cooke (2020) (Table 2). For PCR reaction, Qiagen (MD, USA) Microsatellite PCR kit and 20 to 25 ng template DNA were used and all other PCR reactions and thermal cycling conditions were used as described in Li et al. (2013), whereas concentration of primers was used as described by Cooke (2020). Amplified PCR products were diluted in distilled water to 1:10 ratio, and samples were genotyped at the Sequencing Center of the University of British Columbia, Vancouver, using the ABI 3730 capillary DNA sequencer (Applied Biosystems, CA, USA) according to the manufacturer’s instructions.

**Table 2:** Details of multiplex simple sequence repeat (SSR) analyses in genotyping *Phytophthora infestans* isolates

Locus	†Dye used	Primer concentration (µM) used	Expected sizes of amplicons (bp)	Size of amplicon (bp) in this study	No. of alleles
PiG11	NED	0.070	130–206	134–160	5
Pi02	NED	0.043	255–275	260–270	3
PinfSSR11	NED	0.043	325–360	331–355	3
D13	FAM	0.16	100–210	108–134	4
PinfSSR8	FAM	0.32	250–275	260–270	3
PinfSSR4	FAM	0.052	280–305	284–310	11
Pi04	VIC	0.03	160–175	160–170	4
Pi70	VIC	0.070	185–205	192	2
PinfSSR6	VIC	0.035	230–250	246	2
Pi63	VIC	0.052	265–280	270–279	2
PinfSSR2	PET	0.035	165–180	173–175	2
Pi4B	PET	0.24	200–295	213–225	3

SSR loci and dye were used in SSR analyses as described by Li et al. (2013); primer

concentration was used as described by Cooke (2020). †Four types of dye used are fluorescent labels from Applied Biosystems, Canada

**Fig. 1:**
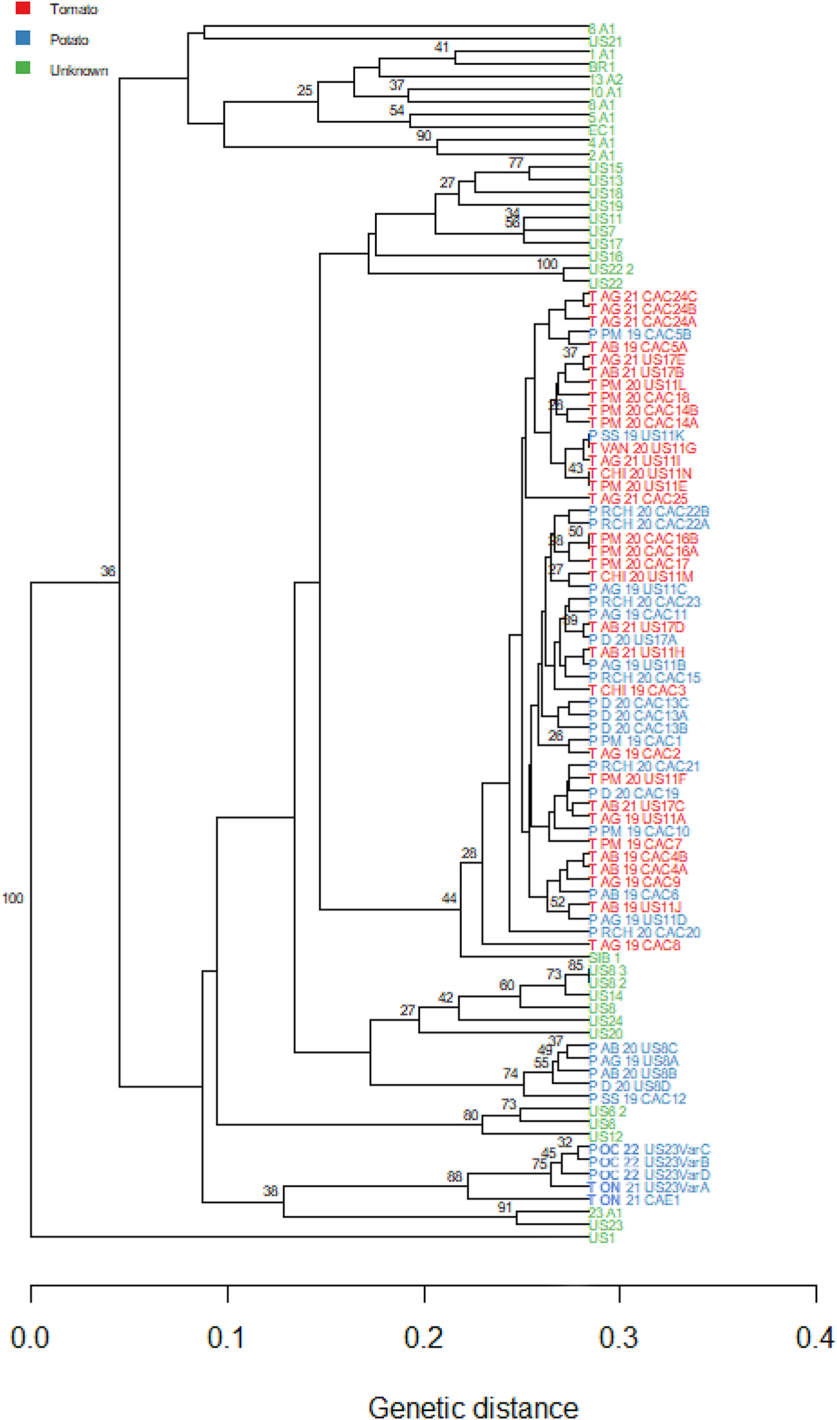
Neighbor-joining phylogram of *Phytophthora infestans* isolates from this study and reference isolates of US and European genotypes of *P*. *infestans* obtained from USABlight (https://usablight.org/identify-ssr-genotype/) and EuroBlight (https://agro.au.dk/forskning/internationale-platforme/euroblight) archives. *P. infestans* isolate codes with green color represent the reference isolates; blue represents British Columbia (BC) and Quebec potato isolates and Ontario (ON) tomato isolates; and red represents BC tomato isolates. Following letters in isolates code mean: US, United States; EC, Ecuador; BR, Brazil; T, tomato; P, potato; AB, Abbotsford; AG, Agassiz; CHI, Chilliwack; D, Delta; PM, Pitt meadows; SS, South Surrey; VAN, Vancouver Island; RCH, Richmond; ON, Ontario; QC Quebec; 19, 2019; 20, 2020; 21, 2021; 22, 2022; CAC, Canada coast; CAE, eastern Canada. AB, AG, CHI, D, PM, SS, VAN, and RCH are locations from BC

The peak size was determined against a GeneScan 500 LIZ standard (Applied Biosystems, CA, USA), and alleles were scored manually using the Peak Scanner v2 (Applied Biosystems, CA, USA) with fragment sizes rounded up to the nearest whole number for analysis. After that, the allelic banding patterns were compared with those of the reference isolates and other published data and assigned their nomenclature accordingly. Ten out of 12 SSR markers produced multiple alleles (2 to 11 alleles), whereas markers Pi04 and PinfSSR6 had 2 alleles (Table 2). For SSR genotyping data, all loci were considered triploid to include the third allele present in those isolates. When a third allele was not present in those individual isolates, it was considered a null allele. The SSR classifier tool (USABlight https://usablight.org/identify-ssr-genotype/, retrieved on 27 December 2022) was used to identify genotypes of isolates. The tool identifies the genotype of isolates by comparing allelic patterns of unknown isolates with reference isolates. Those isolates whose genotypes did not match with known genotypes in the SSR classifier tool at https://usablight.org were assigned as new genotypes (Table 1 and Fig. 1) based on the visual examination of allelic patterns of isolates and Bruvo’s genetic distance used in constructing a neighbor-joining (N-J) tree to visualize the clades (Olave-Achury et al. 2022). Using the Poppr v.2.8.3 (Kamvar et al. 2014) and R library adegenet v.2.1.1 (Jombart 2008), N-J tree was constructed based on Bruvo’s distance and a combination model (genome addition and genome loss). The data set was bootstrapped using 1000 replicates. In the N-J tree, we used *P*. *infestans* isolates from this study (204 isolates from BC, 2 isolates from ON, and 4 isolates from QC, Canada) and 34 reference isolates from the USA, Europe, and South America retrieved from the USABlight archive at https://usablight.org. A genetic distance of 0.28 was used as the cut-off value to determine the exact relatedness of two or more isolates. Nomenclatures of new genotypes were given as “CAC1 to CAC25” and “CAE1” (Table 1).

### Population analyses

Using genotyping data of 12 SSR loci of *P.* *infestans* isolates (*n* = 204) from BC, the genetic diversity and population structure of those isolates were analyzed. *P. infestans* isolates were classified into populations based on host type, location, year, and major genotypes (Table 3). The R library Poppr (Kamvar et al. 2014) was used to calculate population statistics (genetic diversity). The population statistics included the following: MLGs, the number of multilocus genotypes; eMLG, the number of expected MLGs at the smallest sample size of at least 10 (Hurlbert 1971); *H*, Shannon-Weiner index of MLG diversity (Shannon 2001); *G*, Stoddart and Taylor index of MLG diversity (Stoddart and Taylor 1988); *λ*, Simpson index corrected for sample size by multiplying the index value by N/(N-1) (Wang et al. 2017); evenness (Grünwald et al. 2003); *H*_exp_, Nei’s unbiased gene diversity; *I*_A_, index of association (Brown et al. 1980); and *r¯*_*d*_, the standardized index of association which removes sample size bias (Agapow and Burt 2001). Clone-corrected data were used in calculating the index of association (*I*_*A*_) and standardized index of association (*r¯*_*d*_), whereas all data were used to estimate other population diversity indices. R library Poppr v.2.8.1 (Kamvar et al. 2014) was used to generate clone-corrected data, where clonal isolates were removed such that each population contained only one representative of each haplotype.

**Table 3:** Estimates of genetic diversity statistics for *Phytophthora infestans* populations. Populations were classified based on host, year, locations, and genotype groups. Allelic data generated from 12 simple sequence repeat (SSR) loci were used in the genetic study

Population	*N*	MLG	eMLG (SE)	*H*	*G*	*λ*	Evenness	*H*_exp_	*I*_A_	*r¯*_*d*_
Year and host										
2019 Tomato	55	15	9.99 (1.46)	1.94	4.03	0.752	0.511	0.236	− 0.219	− 0.0465
2019 Potato	28	12	12 (0)	1.93	4.31	0.768	0.561	0.266	1.731	0.2672
2020 Tomato	63	16	11.5 (1.42)	2.23	6.37	0.843	0.646	0.272	− 0.113	− 0.0287
2020 Potato	31	15	15 (0)	2.38	7.94	0.874	0.708	0.316	3.275	0.5031
2021 Tomato	27	15	15 (0)	2.5	9.99	0.9	0.806	0.265	− 0.181	− 0.0455
Year										
2019	83	23	11.2 (1.76)	2.20	4.42	0.774	0.425	0.246	1.092	0.1654
2020	94	30	14.2 (1.87)	2.81	10.23	0.902	0.594	0.288	2.206	0.3285
2021	27	15	15.0 (0)	2.50	9.99	0.900	0.806	0.265	− 0.181	− 0.0455
Total	204	56	15.1 (2.12)	3.07	9.16	0.891	0.396	0.271	1.301	0.1964
Location (Pacific western Canada, BC)										
Abbotsford	53	17	6.65 (1.176)	2.39	7.96	0.874	0.703	0.269	2.7122	0.4061
Agassiz	32	19	7.99 (1.099)	2.70	11.13	0.910	0.727	0.269	0.8301	0.1280
Chilliwack	31	7	3.72 (0.990)	1.20	2.22	0.549	0.528	0.247	− 0.1262	− 0.0321
South Surrey	10	4	4.00 (0)	1.09	2.38	0.580	0.701	0.286	2.4737	0.7201
Pitt Meadows	45	20	7.40 (1.181)	2.64	10.18	0.902	0.709	0.273	− 0.2859	− 0.0593
Delta	20	7	5.18 (0.867)	1.67	4.26	0.765	0.755	0.297	3.0704	0.4672
Richmond	7	7	7.00 (0)	1.95	7.00	0.857	1.000	0.276	− 0.0556	− 0.0186
Vancouver	6	1	1.00 (0)	0.00	1.00	0.000	NAN‡	0.286	NAN	NAN
Total	204	56	7.20 (1.340)	3.07	9.16	0.891	0.396	0.271	1.301	0.1964
*Genotype groups										
US11	121	13	4.54 (1.134)	1.698	3.47	0.712	0.554	0.254	− 0.102	− 0.0277
CAC6	5	1	1.00 (0)	0.000	1.00	0.000	NAN	0.245	NAN	NAN
CAC4	10	2	2.00 (0)	0.500	1.47	0.320	0.725	0.219	NAN	NAN
US17	15	5	3.98 (0.756)	1.170	2.42	0.587	0.639	0.238	− 0.131	− 0.1336
CAC13	10	3	3.00 (0)	0.639	1.52	0.340	0.576	0.248	− 0.500	− 0.500
CAC24	6	3	3.00 (0)	0.868	2.00	0.500	0.724	0.247	0.000	NAN
Total	204	56	7.20 (1.340)	3.072	9.16	0.891	0.396	0.271	1.301	0.1964

*N* number of isolates of *P*. *infestans*, *MLG* number of multilocus genotypes (MLG), *eMLG* the number of expected MLG at the smallest sample size of at least 10, *SE* standard error, *H* Shannon–Wiener index of MLG diversity, *G* Stoddart and Taylor’s index of MLG diversity, *λ* Simpson’s index, *H*_*exp*_ Nei’s 1978 gene diversity, *I*_A_ index of association, *r*¯_*d*_ Standardized index of association

^*^Genotypes with less than 5 isolates (*n* = 5) were not presented in the table; NAN‡, not applicable

The population structure of *P*. *infestans* isolates was analyzed using the software Structure Harvester v2.3.3 (Pritchard et al. 2000) and clone-corrected data were used. The data were run using 20,000 burn-in repeats and 1,000,000 Markov Chain Monte Carlo (MCMC) repeats under an admixture model to accommodate samples that may have originated from sexual recombination events. Independent runs of the model used *K* values from 1 to 10 with 20 replicate runs at each value of *K*. The optimal *K* was estimated using the Evanno method in the web tool Structure Harvester (Evanno et al. 2005; Earl and VonHoldt 2012). Also, the optimal *K* was inferred by directly observing the groupings of the samples by their assigned Q values. All runs for optimal *K* and non-optimal *K* values were averaged using CLUMPP v1.1.2 (Jakobsson and Rosenberg 2007) and visualized with the program DISTRUCT v.1.1 (Rosenberg 2004).

In addition, the genetic groupings were visualized by conducting a principal component analysis (PCA) and discriminant analysis of principal components (DAPC) using the R library adegenet (Jombart 2008; Beninal et al. 2022). PCA was constructed using the SSR data and isolates were grouped based on a combination of location, sampling year and host, whereas DAPC was constructed using SSR data from only BC to detect variation in BC isolates. To understand the variability in *P*. *infestans* populations between hosts, and among years, locations, and genotypes, a minimum spanning network (MSN) was constructed for samples from BC (*n* = 204), ON (*n* = 2), and QC (*n* = 4) based on Bruvo’s genetic distance using R library adegenet v2.1.1 (Jombart 2008) in the R package Poppr (Kamvar et al. 2014).

### Mating type and allozyme analyses

Mating types of the representative isolates of *P. infestans* (*n* = 66) were determined using the protocol described by Alkher et al. (2015). Strains US24 (A1) and US8 (A2) previously characterized by Peters et al. (2014) were used as tester strains. In brief, isolates forming oospores on plates paired with the A1 mating type (US24) were designated as the A2 mating type, while those that formed oospores on plates paired with the A2 mating type (US8) were designated as the A1 mating type.

Representative isolates of *P. infestans* (*n* = 56) were examined at the *Gpi* locus using cellulose acetate electrophoresis (CAE) to determine allozyme genotypes. The isolates were grown on pea agar (Peters et al. 2014) for 2 weeks, and mycelia were scrapped from the plates and placed in a 1.5-mL Eppendorf microcentrifuge tube and processed as described by Goodwin et al. (1995a) and Peters et al. (2014). Reference isolates (US 8, US11, US 22, US23, and US24) were included in the run, and the migration distances of Gpi proteins from the reference genotypes were compared with those from the uncharacterized isolates.

### In vitro sensitivity to metalaxyl-m

Metalaxyl-m sensitivity of the *P. infestans* isolates (*n* = 183) was determined using technical grade metalaxyl-m (Syngenta, Basel, Switzerland). Two experiments were conducted with three replicates in each experiment and the data were pooled for analysis. Metalaxyl-m sensitivity assays were conducted using methods described previously (Peters et al. 2014; Alkher et al. 2015). Briefly the stock solution of metalaxyl-m was prepared as 100 mg ml^−1^ in dimethyl sulfoxide (DMSO). Final concentrations of 0, 1, 10, and 100 µg ml^−1^ by amending stock solution to clarified pea agar. Radial growth of each isolate was measured at 14 days after incubation. Isolates were categorized as: metalaxyl sensitive (MS), which had < 10% growth; metalaxyl moderately resistant (MMR), which had 10–60% growth; and metalaxyl highly resistant (MHR), which had > 60% growth at 100 µg ml^−1^ of metalaxyl-m.

## Results

### Prevalence of late blight and pathogen isolation

Late blight was found in BC in all 3 years (2019 to 2021); however, disease incidence timing and severity varied greatly over the years. Late blight was found in several commercial farms, research and small farms, and home and community gardens of eight diverse locations in BC (Table 1). In 2019, the disease was observed in field-grown tomatoes and potatoes on small farms in Chilliwack and Abbotsford during the second week of September and late blight severity was very high (~ 60 to 100%). During mid-September to the first week of October, the disease was also found in tomatoes and potatoes at several community and home gardens in Agassiz, Chilliwack, Abbotsford, and Pitt Meadows. Disease severity was also high (~ 40 to 50%) in three locations and completely wiped out in most of the plots in Pitt Meadows. In addition, late blight was found in a few patches of potato fields of the Agassiz research farm and commercial farms in Cloverdale, BC, in late September and early October; the limited spread of the disease in these plots could be due to routine application of fungicides. In 2020, late blight symptoms were observed early in the season (between mid-July and mid-August) in several commercial potato farms in BC (*n* = 10) including Delta, Richmond, Surrey, and Abbotsford. Late blight was found in a few patches of these fields with low to moderate disease severity (~ 15 to 25%). Late blight symptoms were also observed in tomatoes grown at several communities and home gardens in the Fraser Valley regions including Chilliwack, Abbotsford, and Pitt Meadows in August and September. Late blight severity in these communities and home gardens was high (~ 40% to 100%). In 2021, late blight symptoms were not found in commercial potato farms, but the disease was observed late in the season (early to mid-October) in community gardens in Abbotsford and Agassiz. Late blight was not found in most of the locations from BC in 2022, but the disease was reported in few farms in Delta. A total of 204 isolates of *P. infestans* (83 in 2019, 94 in 2020, and 29 in 2021) were obtained from pathogen-infected samples of potatoes (*n* = 57) and tomatoes (*n* = 147) from 28 fields in 8 locations in BC during 2019–2021 (Table 1). *P. infestans* cultures were isolated from infected leaves and stems of tomato and potato and fruits of tomato.

In contrast, late blight incidence was very low or absent in other provinces of Canada during 2019–2022 due to the unfavorable climatic conditions (persistent high temperature, low humidity, and dry weather) during the growing seasons (data not shown). However, the disease was sporadic in eastern Canada and two isolates of *P*. *infestans* were obtained from tomato samples from ON in 2021 and four isolates from potato samples were obtained from QC in 2022.

### Identification of pathogen genotypes

Genotypes were identified directly from six infected tissue samples processed on FTA cards as well as DNA extracted from pure cultures of 210 isolates. Twelve SSR markers were able to amplify six samples processed directly on FTA cards and identified two genotypes, US11 (*n* = 5) and US17 (*n* = 1) of *P*. *infestans*. Among the SSR loci of 210 isolates, SSR4 had the highest number of alleles (*n* = 11), and Pi70 and SSR6 had the least number of alleles (*n* = 2) (Table 2). Among six potato isolates from eastern Canada, 5 were of US23 genotype and one isolate was a new genotype (CAE1). Among 204 isolates of *P*. *infestans* in BC, we identified US8 (3%), US11 (59%), US17 (7%), and 25 new genotypes “CAC1 to CAC25” (30%) (Table 1). The details of allelic patterns of 12 SSR makers of all genotypes were provided in Supplemental Table S1. Frequency distribution of pathogen genotypes varied with host, location, and year of collection. US8 was exclusively found in potato samples and only from three locations. US17 was detected for the first time in BC, and this genotype was found in both hosts and in multiple locations and years at low frequencies. US11 was the most dominant genotype across hosts, years, and locations. Approximately 67% of tomato isolates and 35% of potato isolates were of the US11 genotype. The frequency of US11 was very high in tomato samples collected from home and community gardens of Chilliwack (94%), Pitt Meadows (67%), and Vancouver Island (100%). The frequency of US11 was also high across years in these locations. In 2021, we only isolated *P*. *infestans* from tomato foliage from community gardens of Agassiz (*n* = 15) and Abbotsford (*n* = 12), where 58% of the isolates in Agassiz were new genotypes and 80% of the isolates from Abbotsford were US11. We discovered 25 new genotypes in BC, whose SSR banding patterns did not match with previously known genotypes. Phylogenetic analyses showed that most of these new genotypes were genetically close to US11, and a few of them were close to US17 and US8 (Fig. 1). A few new genotypes were in distinct clusters. New genotypes CAC5, CAC6, and CAC11 were recovered from both potato and tomato samples, whereas the other genotypes were found exclusively on either potatoes or tomatoes (Table 1 and Supplemental Table S1). Only a few new genotypes (CAC2 and CAC24) were detected from multiple locations, whereas the vast majority of them (> 95%) were only found in a single location and in the same year. The highest frequency of new genotypes in tomato samples was found in Abbotsford in 2019 (63%), followed by Agassiz in 2021 (58%), whereas the highest frequency in potato samples was observed in commercial farms in Delta (55%) and Richmond (86%).

### Genetic diversity

High genetic diversity was found in *P. infestans* populations categorized based on host, location, and year (Table 3). Overall, 56 MLGs were detected from BC populations (*n* = 204) and five MLGs from isolates from eastern Canada (*n* = 6). Among the populations based on host and year, the 2021 tomato had the highest MLGs and diversity indices and closely followed by the 2020 potato population (Table 3). High values of *H*, *G*, *λ*, evenness, and *H*_exp_ were found in populations categorized based on the host in each year (Table 3). Among years, the 2019 population showed slightly lower numbers of MLGs (23 out of 83 isolates) than the populations from 2020 (30 out of 65) and 2021 (15 out of 27). Similar patterns of other genetic diversity indices (*H*, *G*, *λ*, evenness, and *H*_exp_) were observed between the 2020 and 2021 populations, whereas the 2019 population had slightly lower indices of genetic diversity than the other 2 years (Table 3). Among locations, Abbotsford (17 out of 53 isolates), Agassiz (19 out of 32), and Pitt Meadows (20 out of 45) had higher MLGs than other locations. Values of *H*, *G*, *λ*, evenness, and *H*_exp_ were also high in the pathogen populations from these locations (Table 3). Among populations categorized based on genotype group, the US11 genotype had the highest genetic diversity indices (*H* = 1.698, *G* = 3.47, and *λ* = 0.712) (Table 3).

Values of *I*_A_ and *r¯*_*d*_ were further away from zero in potato population (*I*_A_ = 3.275 and 1.731 for 2020 and 2019, respectively), indicating a greater level of clonality, whereas these values were close to zero in tomato populations, indicating a greater level of recombination. These values were close to zero in populations from Chilliwack and Pitt Meadows and most of the isolates from these locations were from tomatoes. Within a potato population, isolates obtained from Delta had an* I*_A_ furthest from 0 (*I*_A_ = 3.0704), indicating the greatest level of clonality, whereas isolates from Richmond showed the greatest level of recombination (*I*_A_ =  − 0.0556 and *r¯*_*d*_ =  − 0.0186) (Table 3).

### Population structure

The N-J tree distinguished the European and American isolates by separating them into two major clades. Isolates from this study that matched with known genotypes (US8, US11, US17, and US23) clustered with corresponding genotypes. For example, the US23 reference isolates clustered with those obtained from eastern Canada and the US8 isolates from BC formed a clade with the US8 reference isolate. A few isolates from our study either formed a distinct cluster or clustered with either US8 or US17, whereas the majority of the new isolates clustered with US11 (Fig. 1).

**Fig. 2:**
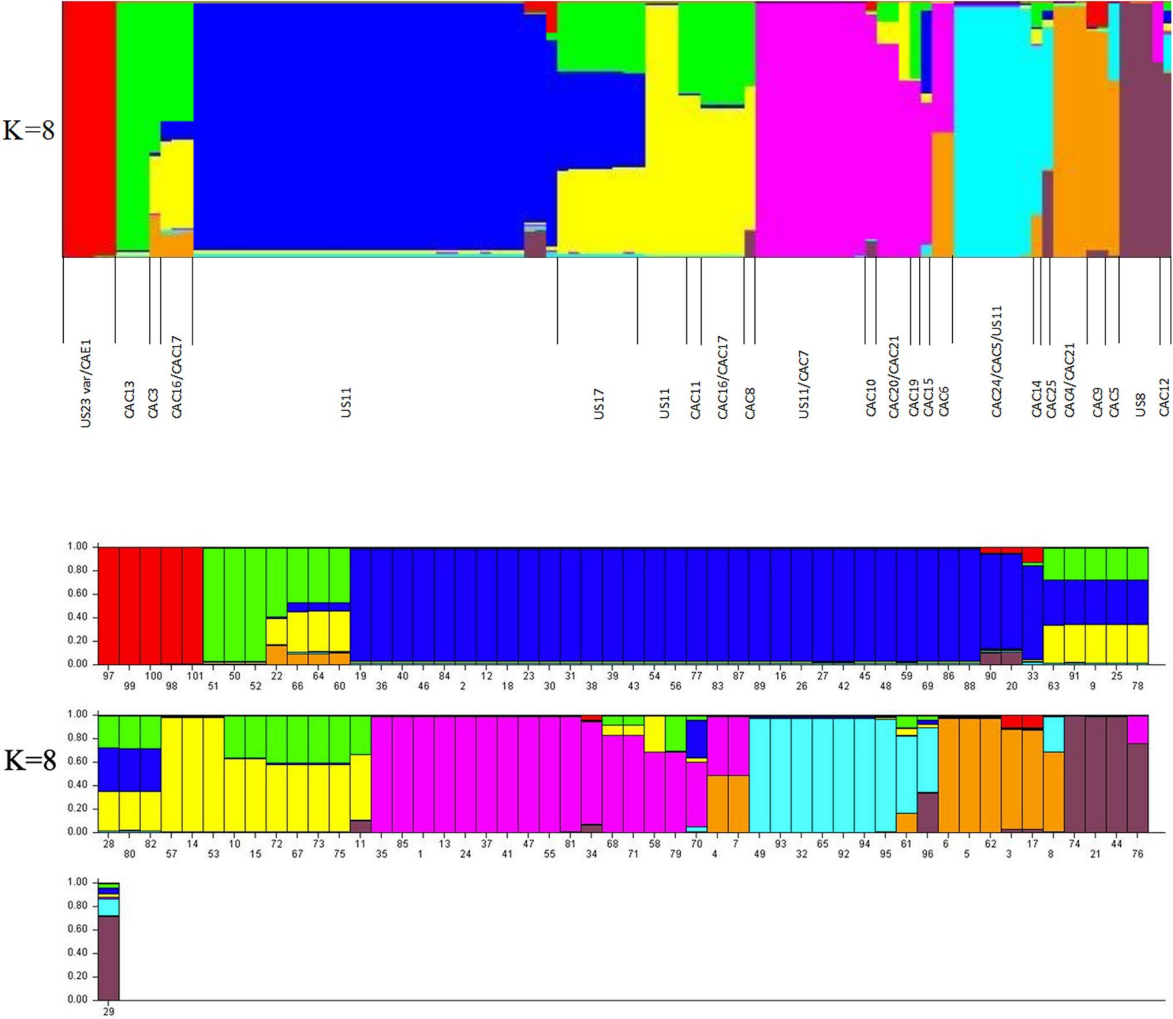
Structure analysis of *Phytophthora infestans* isolates from British Columbia, Ontario, and Quebec using allelic data generated from 12-plex simple sequence repeats (SSR) loci. The graph was generated from a single run of structure (*K* values 1 to 10; burn-in chain of 20,000 repeats; Markov chain Monte Carlo of 1,000,000 repeats; each *K* value repeated 20 times)

Population structure analysis using Structure harvester determined the optimal *K* value of 8 based on the Evanno method (Fig. 2). At *K* = 8, the population clustered based on the identified genotypes. Isolates (US23 var and CAE) from ON and QC clustered together. Most of the isolates identified as the US11 formed a distinct cluster with slight subclonal variations among the US11 isolates. Isolates of US17 and US8 formed separate clusters, respectively (Fig. 2). New genotypes CAC11 and CAC22 formed another cluster and grouped closely with the US11 isolates (Fig. 2). Isolates belonging to the new genotypes CAC13, CAC14, CAC16, and CAC17 formed separate individual clusters. Likewise, new genotypes CAC20 and CAC21 formed a cluster (Fig. 2). In contrast, new genotypes CAC3, CAC12, CAC15, CAC10, CAC25, CAC5, CAC8, CAC1, and CAC19 are distinct individually and no clusters formed.

**Fig. 3:**
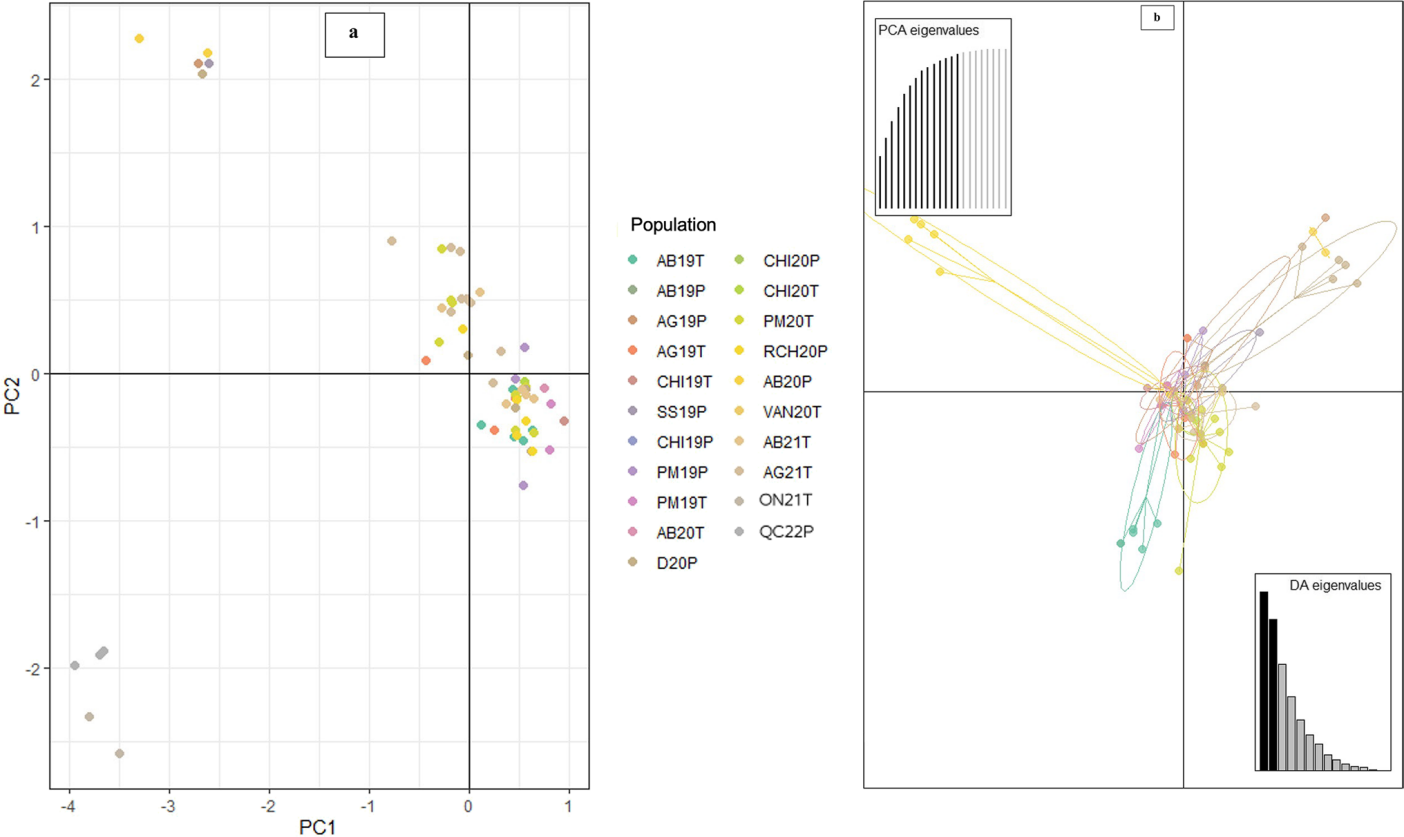
**a** Principal component analysis (PCA) plot of *Phytophthora infestans* isolates from British Columbia, Ontario, and Quebec using allelic data generated from 12-plex simple sequence repeats (SSR) loci*.* All isolates from this study were included. T, tomato; P, potato; AB, Abbotsford; AG, Agassiz; CHI, Chilliwack; D, Delta; ON, Ontario; QC, Quebec; PM, Pitt meadows; SS, South Surrey; VAN, Vancouver Island; RCH, Richmond; and 19, 2019; 20, 2020; 21, 2021; and 22, 2022. **b** Discriminant analysis of principal components (DAPC) plot of *P. infestans* isolates from British Columbia

PCA revealed that *P. infestans* isolates from ON and QC (ON21T and QC22P) representing the genotypes US23 and CAE1 clustered together and were distinct from other genotypes (Fig. 3a). Likewise, BC isolate groups AB20P, AG19P, SS19P, and D20P consisting of the US8 and CAC12 formed a separate cluster, while the remaining isolates from BC including the US17, US11, and other novel isolates clustered together with varying subclonal variations observed among the groups (Fig. 3a). DAPC showed that the US17 and other new genotypes from Agassiz and Abbotsford in 2019 and 2021 from potato and tomato formed a distinct cluster (Fig. 3b). Another distinct cluster was formed by isolates from Abbotsford in 2019, alongside isolates originating from tomato samples in Pitt Meadows in 2020 (Fig. 3b).

**Fig. 4:**
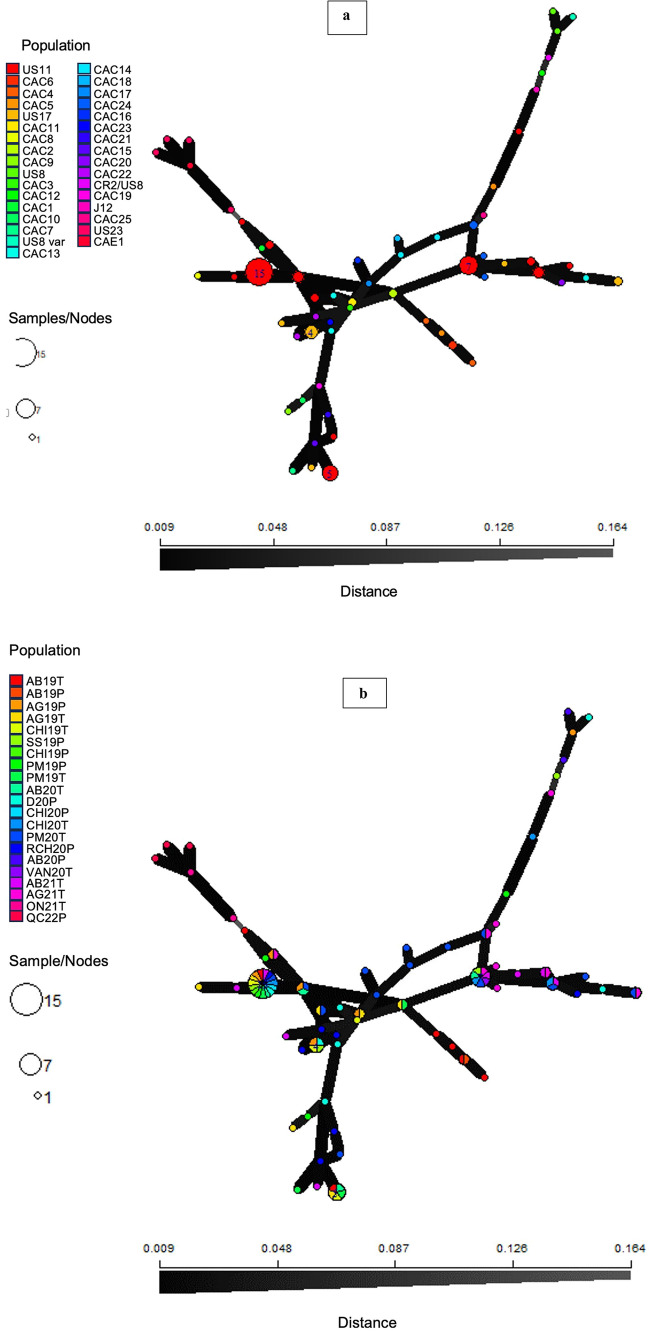
**a** Minimum spanning network (MSN) tree of *Phytophthora infestans* isolates from British Columbia, Ontario, and Quebec using allelic data generated from 12-plex simple sequence repeats (SSR) loci. Bruvo’s distance was used to generate MSN tree based on genotypes of *P*. *infestans*. Node size is proportional to the number of isolates of *P. infestans*. US, United States; CAC, Canada coast; CAE, eastern Canada. **b** MSN tree of *P. infestans* isolates from British Columbia, Ontario, and Quebec. Bruvo’s distance was used to generate MSN tree based on a combination of location, year, and host. The letters on the figure artwork indicate the following: T, tomato; P, potato; AB, Abbotsford; AG, Agassiz; CHI, Chilliwack; D, Delta; ON, Ontario; QC, Quebec; PM, Pitt meadow; SS, South Surrey; VAN, Vancouver Island; RCH, Richmond; 19, 2019; 20, 2020; 21, 2021

**Fig. 5:**
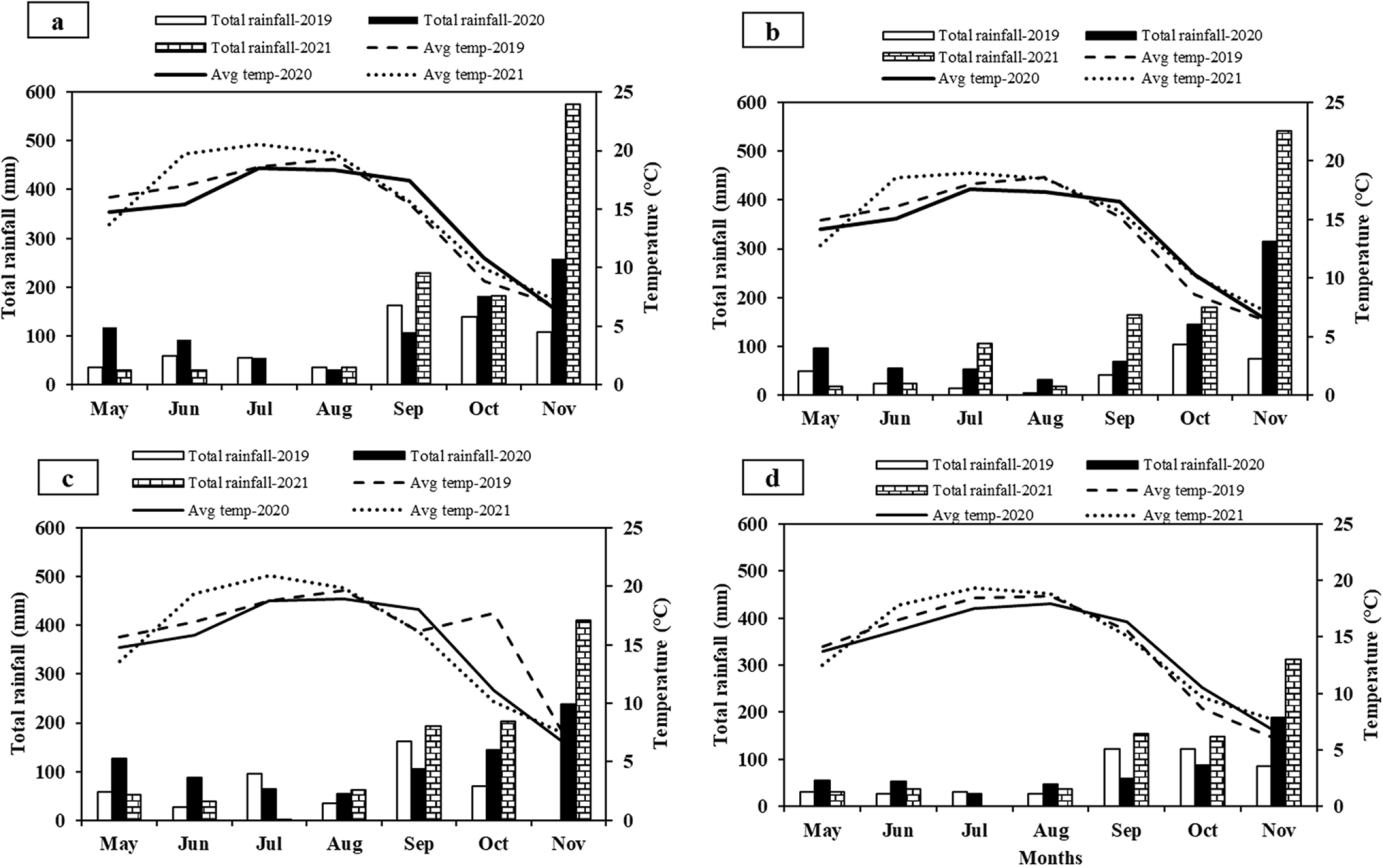
Monthly total rainfall (mm) and average temperature (°C) from May to November between 2019 to 2021 at field sites at Agassiz (**a**), Abbotsford **(b)**, Pitt Meadow (**c**), and Vancouver (**d**) in British Columbia, Canada

MSN showed the US8, CAC12, and J12 (AB20P, AG19P, SS19P, and D20P) isolates clustered together to form a unique cluster on the same branch on MSN (Figs. 5a and b). Also, *P. infestans* isolates from ON and QC (ON21T and QC22P) comprising the US23 and CAE1 formed a distinct branch. The US17 isolates clustered together and formed a branch (Fig. 4a and b) while CAC24 and CAC25 were located close to the center of the MSN as was CAC13. The clustering observed using the MSN is similar to those observed for other clustering analyses, but the MSN revealed more subclonal variations in US11 and some of the novel genotypes (Fig. 4a and b).

### Mating type and allozyme analyses

*P.* *infestans* isolates from ON and QC (*n* = 6) and BC (*n* = 60) were of the A1 mating type. These include the US11, US17, US23, and a few of the new genotypes (Table 4). Allozyme analyses showed two Gpi banding pattern types were observed across the sampling years and locations consisting of the Gpi 100/100/111 and Gpi 100/100 (Goodwin et al. 1995a) belonging to the US11, US17, US23, and some new genotypes (Table 4). Overall, *P. infestans* isolates from BC banded as either 100/100/111 or 100/100 whereas all isolates from QC and ON banded as 100/100.

**Table 4:** Glucose-6-phosphate isomerase (Gpi) allozyme banding patterns and mating type of each genotype of *Phytophthora infestans* isolates

Genotype	Allozyme analysis		Mating type analysis	
	No of isolates	Gpi banding pattern	No of isolates	Mating type
US11	23	100/100/111	34	A1
	9	100/100		
US17	3	100/100/111	6	A1
US23 var	1	100/100	5	A1
CAC1	1	100/100/111	1	A1
CAC4	3	100/100/111	6	A1
	6	100/100		
CAC5	1	100/100/111	1	A1
CAC6	3	100/100/111	3	A1
CAC7	-	-^b^	1	A1
CAC9	-	-	1	A1
CAC10	1	100/100/111	1	A1
CAC11	2	100/100	-	-
CAC12	1	100/100	-	-
CAC24	-	-	5	A1
CAC25	-	-	1	A1
CAE1	1	100/100	1	A1
Total	**56**		**66**	

^a^Glucose-6-phosphate isomerase, ^b^Not tested, genotype names initiated with letter

“CAC” are new genotypes. *US* United States, *CAC* Canada coast, *CAE* eastern Canada

In vitro sensitivity to metalaxyl-m.

About 95% of *P*. *infestans* (*n* = 181) originating from BC between 2019 and 2021 were resistant (MMR or MHR) to metalaxyl-m (Table 5). The frequency of metalaxyl-resistant isolates was high across genotypes, years, and locations, whereas the US23 and CAE1 isolates (*n* = 2) originating from both ON and QC were sensitive to metalaxyl (Table 5).

**Table 5:** Source and genotypes of *Phytophthora infestans* and their sensitivity to metalaxyl-m in *in vitro* studies. *MS* metalaxyl sensitive, *MMR* metalaxyl moderately resistant, *MHR* metalaxyl highly resistant

Genotype	Location	Host	2019 strains				2020 strains				2021 strains			
			*n*	MS	MMR	MHR	*n*	MS	MMR	MHR	*n*	MS	MMR	MHR
US11	Abbotsford	Tomato	8	0	5	3	11	0	1	10	10	0	5	5
	Agassiz	Tomato	6	0	5	1	-	-	-	-	3	0	3	0
	Agassiz	Potato	5	1	4	0	-	-	-	-	-	-	-	-
	Chilliwack	Tomato	12	0	10	2	12	0	1	11	-	-	-	-
	Chilliwack	Potato	2	0	1	1	1	0	0	1	-	-	-	-
	Cloverdale*	Potato	7	0	6	1	-	-	-	-	-	-	-	-
	Delta	Potato	-	-	-	-	2	0	0	2	-	-	-	-
	Pitt Meadows	Tomato	4	0	1	3	21	0	5	16	-	-	-	-
	Richmond	Potato	-	-	-	-	1	0	0	1	-	-	-	-
Total			**44**	**1**	**32**	**11**	**48**	**0**	**7**	**41**	**13**	**0**	**8**	**5**
US17	Abbotsford	Tomato	-	-	-	-	-	-	-	-	3	0	3	0
	Agassiz	Tomato	-	-	-	-	-	-	-	-	2	0	1	1
	Agassiz	Potato	3	1	1	1	-	-	-	-	-	-	-	-
	Chilliwack	Tomato	1	0	1	0	-	-	-	-	-	-	-	-
	Cloverdale	Potato	2	1	1	0	-	-	-	-	-	-	-	-
	Delta	Potato	-	-	-	-	3	0	3	0	-	-	-	-
	Pitt Meadows	Tomato	-	-	-	-	1	0	0	1	-	-	-	-
Total			**6**	**2**	**3**	**1**	**4**	**0**	**3**	**1**	**5**	**0**	**4**	**1**
US8	Agassiz	Potato	1	1	0	0	-	-	-	-	-	-	-	-
	Abbotsford	Potato	-	-	-	-	3	0	2	1	-	-	-	-
	Delta	Potato	-	-	-	-	3	1	1	1	-	-	-	-
Total			**1**	**1**	**0**	**0**	**6**	**1**	**3**	**2**	**-**	**-**	**-**	**-**
US23 var	Ontario	Tomato	-	-	-	-	1	1	0	0	-	-	-	-
CAC1	Pitt Meadows	Potato	1	0	1	0	-	-	-	-	-	-	-	-
CAC2	Agassiz	Tomato	1	0	1	0	-	-	-	-	-	-	-	-
	Pitt Meadows	Tomato	1	0	1	0	-	-	-	-	-	-	-	-
CAC4	Abbotsford	Tomato	10	1	7	2	-	-	-	-	-	-	-	-
CAC5	Abbotsford	Tomato	1	0	1	0	-	-	-	-	-	-	-	-
	Pitt Meadows	Potato	1	0	1	0	-	-	-	-	-	-	-	-
CAC6	Abbotsford	Tomato	3	0	2	1	-	-	-	-	-	-	-	-
	Abbotsford	Potato	1	0	1	0	-	-	-	-	-	-	-	-
CAC7	Pitt Meadows	Tomato	1	0	1	0	-	-	-	-	-	-	-	-
CAC8	Agassiz	Tomato	1	0	1	0	-	-	-	-	-	-	-	-
CAC9	Agassiz	Tomato	1	0	1	0	-	-	-	-	-	-	-	-
CAC10	Pitt Meadows	Potato	1	0	1	0	-	-	-	-	-	-	-	-
CAC11	Agassiz	Potato	1	0	0	1	-	-	-	-	-	-	-	-
	Agassiz	Tomato	1	0	0	1	-	-	-	-	-	-	-	-
CAC12	Cloverdale	Potato	1	0	1	0	-	-	-	-	-	-	-	-
CAC13	Delta	Potato	-	-	-	-	9	2	4	3	-	-	-	-
CAC14	Pitt Meadows	Tomato	-	-	-	-	2	0	1	1	-	-	-	-
CAC15	Richmond	Potato	-	-	-	-	1	0	0	1	-	-	-	-
CAC16	Pitt Meadows	Tomato	-	-	-	-	2	0	1	1	-	-	-	-
CAC17	Pitt Meadows	Tomato	-	-	-	-	1	0	1	0	-	-	-	-
CAC18	Pitt Meadows	Tomato	-	-	-	-	1	0	1	0	-	-	-	-
CAC19	Delta	Potato	-	-	-	-	1	0	1	0	-	-	-	-
CAC20	Richmond	Potato	-	-	-	-	1	0	0	1	-	-	-	-
CAC21	Richmond	Potato	-	-	-	-	1	0	0	1	-	-	-	-
CAC22	Richmond	Potato	-	-	-	-	1	0	1	0	-	-	-	-
CAC23	Richmond	Potato	-	-	-	-	1	0	0	1	-	-	-	-
CAC24	Pitt Meadows	Tomato	-	-	-	-	1	0	0	1	-	-	-	-
	Agassiz	Tomato	-	-	-	-	-	-	-	-	5	0	1	4
CAC25	Agassiz	Tomato	-	-	-	-	-	-	-	-	1	0	1	0
CAE1	Ontario	Tomato	-	-	-	-	-	-	-	-	1	1	0	0
Total			**26**	**1**	**20**	**5**	**22**	**2**	**10**	**10**	**7**	**1**	**2**	**4**
Grand Total			**77**	**5**	**55**	**17**	**81**	**4**	**23**	**54**	**25**	**1**	**14**	**10**

-, no isolates were recovered; *Cloverdale (South Surrey); *n* Number of isolates, *US* United States, *CAC* Canada coast, *CAE* eastern Canada; genotype names initiated with letter “CAC” are new genotypes

## Discussion

Late blight caused by *P. infestans* continues to impact the yield and marketability of potato and tomato in North America and elsewhere, as there has been an increase in disease severity, incidence, and pathogen diversity mainly because of the introduction of new and emerging genotypes (Alkher et al. 2015; Kalischuk et al. 2012; Hu et al. 2012; Fry et al. 2013). These new genotypes often pose disease management challenges because new genotypes may have different phenotypic traits, such as increased virulence and increased insensitivity to commonly used fungicides. In this context, we conducted a comprehensive study to monitor genotypes of *P*. *infestans* from tomato and potato in Pacific western Canada. Furthermore, we examined their genetic diversity and population structure and also compared these isolates with a few isolates from eastern Canada.

In the Pacific western Canada, we found a wide variability in first symptoms occurrence and intensity of late blight among multiple years and locations, which was associated with the variability in climate patterns across years. Late blight occurred on potato and tomato plants grown in home and community gardens in several locations of the Fraser Valley areas in 2019 and 2020 after mid-September, indicating that home and community gardens could be good reservoirs of *P*. *infestans*. Amount and frequencies of rainfall were high in the fall in both years, which afforded a conducive environment for late blight occurrence (Fig. 5). Interestingly, frequent rainfall occurred in summer months (June and July) in 2020, which led to early onset of late blight in several commercial potato farms in BC. In 2020, the greatest precipitation period was approximately 107 mm coupled with prolonged damp weather conditions characterized by high humidity favorable for *P. infestans* proliferation (Fig. 5). This is reflected in the number of isolates obtained in 2020 particularly because of high, multiple late blight incidence rates across BC. In contrast, a prolonged drought occurred from mid-June to late October in 2022 and 2021; therefore, a little incidence of late blight was found in 2022 in early summer, where the disease was found only after mid-October in 2021 in community gardens from two locations. In eastern Canada, dry, hot, and less humid conditions persisted (data not shown) resulting in less or no late blight incidences.

Over the years, various biochemical and molecular markers were used to characterize *P. infestans* populations in Canada and elsewhere to monitor the rapid displacement of common genotypes. More recently, microsatellite markers have been used in Canada (Peters et al. 2014; Alkher et al. 2015), extensively in the USA (Danies et al. 2013; Saville et al. 2016; Saville and Ristaino 2019, 2021) and elsewhere (Dey et al. 2018; Shimelash and Dessie 2020; Saville et al. 2021; Olave-Achury et al. 2022) to identify the genotypes of *P. infestans* populations as well as to study population genetic diversity. Multiplex microsatellite genotyping technique made it feasible to rapidly process a large number of isolates. In addition to genotyping several hundreds of *P*. *infestans* isolates, we also demonstrated that the pathogen genotypes can be identified directly from pathogen-infected potato leaf samples. FTA cards were used for genotyping *P. infestans* in the United States (Saville and Ristaino 2019, 2021), in Europe (Li et al. 2013; Mabon et al. 2021; Saville et al. 2021; Puidet et al. 2022), and Africa (Njoroge et al. 2019; Beninal et al. 2022). The use of FTA cards will help to eliminate the regulatory hurdle in importing pathogen live cultures from foreign countries and facilitate international collaborations. In addition, the time-consuming and laborious pathogen isolation is not required if genotyping pathogens directly from infected tissues processed on FTA cards performed well (Guichoux et al. 2011; Li et al. 2013). However, pathogen contamination and the amount of pathogen DNA could be issues in this technique. We also used allozyme analyses of (Gpi banding pattern) of representative isolates of *P*. *infestans*. Compared to the SSR allelic pattern, there was less variability in the Gpi banding pattern among isolates indicating that allozyme analyses are not sufficient to identify the genotypes of *P*. *infestans*.

We identified four known genotypes (US8, US11, US17, and US23) and 25 new genotypes of *P*. *infestans* in BC, Pacific western Canada, and frequency distributions of these genotypes varied among years, locations, and hosts (Table 1). The US11 genotype continued to dominate the BC population as we found approximately 60% of the isolates were of this genotype. The US11 genotype was reported in BC between 1994 and 1998 and 2011 and 2012 and in ON from 1996 to 1998 and 2011 (Punja et al. 1998; Peters et al. 1999, 2014; Daayf et al. 2000; Platt et al. 2002; Alkher et al. 2015). In the USA, US11 was reported in Washington in 1994 and 1996–1997, California and New York in 1995 (Goodwin et al. 1998; Dorrance et al. 1999), as well as in Taiwan from 1997 to 2002 (Jyan et al. 2004). Punja et al. (1998) and Peters et al. (1999) reported BC11 with similarities to US11 as the most prevalent genotype in 1995 and 1996 with 91% frequency. BC11 was the only genotype recovered early in the season in BC in 1997 (Punja et al. 1998). More recently, in 2012, US11 with intermediate resistance to metalaxyl, designated as an A1 mating type with 100/100/111 Gpi banding pattern, was reported in BC (Goodwin et al. 1998; Alkher et al. 2015). In our study, only one isolate out of 103 isolates of US11 genotype was sensitive to metalaxyl-m, while 102 isolates were resistant. Notably, US11, an important genotype in BC and some states in the USA was hypothesized as a more fit and competitive sexual recombinant of *P. infestans* (Goodwin et al. 1998; Fry 2020; Gavino et al. 2000; Peters et al. 2014; Saville and Ristaino 2019). This might be responsible for the displacement of existing strains such as US23 previously reported in BC in 2011 and the continued existence and predominance of US11 in BC. Furthermore, considering the close proximity of the sampled locations in the current study to AB and Washington State, there is a likelihood of transfer of inoculum and possible migration of this genotype through transportation of infected potato seed tubers and tomato transplants from other Canadian provinces and neighboring American states where US11 had been consistently reported (Saville and Ristaino 2019). This is not unexpected as there have been similar cases of the introduction of new genotypes through transportation of infected tubers and tomato transplants within and into Canada (Kawchuk et al. 2011; Kalischuk et al. 2012; Peters et al. 2014) and in the United States (Fry et al. 1992, 2013; Goodwin et al. 1994, 1998; Fry 2020; Gavino et al. 2000).

Interestingly, we reported US17 for the first time in BC and Canada and recovered from both potato and tomato samples across multiple locations during 2019–2021. The US17 genotype was designated as A1 mating type which is highly resistant to metalaxyl (Goodwin et al. 1998). We also found all isolates of US17 were A1 mating type and 87% of isolates (*n* = 15) were resistant to metalaxyl in our study. The genotype was previously reported in the USA as a probable sexual recombinant between US6 and US8 and was isolated from tomatoes in Alabama, Florida, New Jersey, and New York during 1996 (Goodwin et al. 1998). The low frequency of the US8 genotype (3%) was recovered from two locations of BC in this study exclusively from potato samples in 2019 and 2020. This might indicate the host specificity of the genotype and correlates with those reported by Saville and Ristaino (2019) and Hu et al. (2012) in which host specificity was observed in some genotypes such as US14 specificity to potato and US21 exclusively isolated from tomato samples and loss or gain of avirulence genes was hypothesized for the observed host-specificity (Gilroy et al. 2011; Vleeshouwers et al. 2011). Previously, US8 was reported in BC from Creston in 1997 (Punja et al. 1998) and in New Brunswick (NB) and Prince Edward Island (PEI) from 2009 to 2010 (Kalischuk et al. 2012), and also in PEI and QC in 2011 (Peters et al. 2014). In addition, US8 was reported in neighboring states in the USA (Washington and California) until 2016 (Saville and Ristaino 2019). The US8 was the dominant genotype in Canada in 1996 apart from BC where the US11 dominated (Peters et al. 1998). The presence of US8 genotypes in our study indicated that this genotype has the ability to adapt to the microclimate of the Pacific Northwest. Only a few isolates of the US23 genotype were recovered from tomato and potato samples from eastern Canada in this study between 2021 and 2022. This genotype was previously reported in BC, AB, Saskatchewan (SK), Manitoba (MB), and NB between 2009 and 2012 (Kalischuk et al. 2012; Peters et al. 2014; Alkher et al. 2015). During this period, US23 displaced previously dominating genotypes of *P*. *infestans* from most of the provinces of Canada except BC (Peters et al. 2014). US23 was also predominated *P. infestans* population in Wisconsin between 2009 and 2010 and in Maryland, Virginia, and Pennsylvania in 2009 (Hu et al. 2012; Gevens and Seidl 2013). Although US23 has remained the predominant genotype in eastern Canada in recent years and the genotype was also reported in BC at low frequency in 2009 and 2010 (Alkher et al. 2015), we did not find any isolates of US23 from BC in our comprehensive survey between 2019 and 2022 (*n* = 204). The continued dominance of US23 in eastern Canada is most likely due to greater infection efficiency, more resilience to overwintering conditions, greater sporulation rates, and greater epidemic potential than other US lineages (Kalischuk et al. 2012; Fall et al. 2015; Seidl Johnson et al. 2015; Saville and Ristaino 2019). However, we do not know why the US23 genotype is absent in BC. The presence of three major genotypes (US11, US8, and US17) and the continued predominance of US11 in BC suggest local adaptation of these genotypes. Microclimate and cultivar type could have played a major role. However, in this study, we did not examine the impact of cultivar types or varieties on *P. infestans* genetic composition and diversity. In addition, changes to the pathogen population in the sensitivity of the fungicides could impact their fitness and competitive advantages to adapt to the local environment (Saville and Ristaino 2019; Matson et al. 2015).

We found 25 new genotypes (approximately 30% of total isolates from this study) of *P*. *infestans* and the majority of them were from tomato samples in community gardens over multiple years. However, new genotypes were also detected from potato samples in commercial potato fields from multiple locations and years. Approximately 94% of isolates of new genotypes (*n* = 53) were resistant to metalaxyl-m. In agreement with our study, new genotypes (such as BC1-BC11) were reported from BC between 1993 and 1997 (Punja et al. 1998) and from ON, QC, and NB (Peters et al. 2001, 2014; Danies et al. 2014; Alkher et al. 2015). Furthermore, new genotypes of *P*. *infestans* were reported from New York, USA (Danies et al. 2014); southern and northern Italy (Saville et al. 2021), and Colombia (Olave-Achury et al. 2022). It is hypothesized that the emergence of these new genotypes in North America could be due to recombination events or mutations accumulated over the years (Punja et al. 1998; Danies et al. 2014; Alkher et al. 2015; Saville and Ristaino 2019), whereas the emergence of the majority of the new isolates in Europe was due to recombination events resulting from sexual reproduction (Anderson et al. 2009; Sujkowski et al. 1994; Drenth et al. 1994; Yuen and Andersson 2013). These new genotypes were often short-lived, localized in a particular region, and only comprised a small percentage of the total samples (Danies et al. 2014; Peters et al. 2014; Alkher et al. 2015; Saville and Ristaino 2019). New genotypes could have better fit and are more competitive (Goodwin et al. 1998; Gavino et al. 2000; Saville and Ristaino 2019). The introduction of genotypes either via migration or evolved naturally in the region could pose new challenges in late blight management due to their genetic composition and phenotypic traits. The severe late bight epidemics in the USA and Canada occurred and caused significant losses for growers and total production abandonment for some tomato growers, which were due to the introduction and distribution of new genotypes from the shipment of tomato transplants to garden centers in retail stores in the northeast United States (Hu et al. 2012; Fry et al. 2013; Alkher et al. 2015; Saville and Ristaino 2019).

PCA, DAPC, Structure, and MSN analyses showed distinct subdivisions and groupings among the large populations of *P. infestans* in BC (Figs. 1, 2, 3, 4, and 5), which is due to variations in their genetic composition. The small number of isolates from eastern Canada was identified as US23, which formed a distinct cluster. Among BC population, US8 isolated exclusively from potato samples, also formed a distinct cluster. Furthermore, genetic sub-structuring was observed within the US11 isolates from various locations in BC and US8 and CAC12 also clustered away from other BC isolates (AB20P, AG19P, SS19P, and D20P). The structure results and other clustering methods used in this study showed an increase in subclonal variation, corroborated by high genetic diversity indices and the number of MLGs within the US11 lineage (Table 3). Similar results were reported for the US23 and US11 lineages in the USA where considerable subclonal variation and recombination were observed in the lineages and within an aggressive Blue 13 lineage in Indian populations (Dey et al. 2018; Saville and Ristaino 2019). Examination of DAPC, PCA, N-J tree, and structure revealed that the novel genotypes from BC shared some genetic similarities with US11 and US17, whereas US8 and US23 shared little genetic similarities and groupings with US11, US17, and the new genotypes. The new genotype CAE1 from eastern Canada clustered with the US23. This shows the close relatedness of these novel genotypes from BC with some known genotypes (US11, US17, and US23). In agreement with our findings, other studies reported that isolates of US23 shared little genetic similarities with other US genotypes but clustered more with isolates from Bolivia, Brazil, and some parts of Mexico. In addition, our phylogram showed the close relatedness of US23 isolated from this study with those obtained from the USA and Europe (23-A1). Previously, US23 had been reported in the USA and Europe (Gevens and Seidl 2013; Stroud et al. 2016; Kröner et al. 2017; Saville and Ristaino 2019). Likewise, the US11 and US17 lineages showed strong genetic similarity levels and clustered together in the phylogram, PCA, DAPC, and MSNs. These isolates share certain similarities including resistance to metalaxyl; both are A1 mating types, identical RFLP, and similar SSR banding patterns. Similar observations were previously made (Hu et al. 2012; Hansen et al. 2016b; Saville and Ristaino 2019).

We observed high genetic diversity among *P*. *infestans* populations in Pacific western Canada as indicated by higher numbers of MLGs and other genetic diversity indices (Table 3). Among genotypes, the US11 genotype had the highest genetic diversity indices, and the 2021 population was the most diverse followed by 2020. Interestingly among locations, Richmond, Agassiz, and Pitt Meadows had higher genetic diversity than other locations. The indices of association results showed that the tomato population was close to a random mating population implying greater potential for recombination, whereas the potato population was away from a random mating population which is a clonal population. In addition, most of the potato isolates were from commercial and research farms and might share common seed sources whereas tomato isolates were mostly from community and home gardens which are from diverse and isolated areas. Other researchers also reported the clonal population of *P*. *infestans* (based on values of indices of association) from Denmark and other Nordic European countries (Brurberg et al. 2011; Montes et al. 2016; Maurice et al. 2019) as well as from the USA and South American countries (Saville and Ristaino 2019). Other studies also reported high genetic diversities in *P*. *infestans* populations from Canada (Chycoski and Punja 1996; Punja et al. 1998; Kalischuk et al. 2012; Peters et al. 2014; Alkher et al. 2015), USA, Mexico, and other countries (Brurberg et al. 2011; Maurice et al. 2019; Saville and Ristaino 2019; Saville et al. 2021). High genetic diversity in BC could be due to mutations and mitotic recombination or gene conversion (Abu-El Samen et al. 2003; Maurice et al. 2019) because *P. infestans* populations in Canada still mainly consist of asexual populations. In our study, an extensive sampling was conducted in BC between 2019 to 2021 and sampling sites include several communities and home gardens from diverse locations with distinct microclimates. Since the pathogen isolates were from several community gardens with multiple plots (~ 40 to 200 micro plots), which could have high diversity in the varieties and types of tomato. For instance, Agassiz had samples from both hosts: potato samples from research farms and tomato samples from community gardens, and likewise, Pitt Meadows had samples from huge community gardens consisting of more than two hundred plots. Also, we had *P. infestans* isolates from infected tomato samples belonging to Roma and Cherry varieties. Therefore, there could be microclimatic variation and diversity in hosts and variety types. Transportation of infected plant materials (seed tubers and tomato transplants) can introduce new genotypes, which could also contribute to genetic diversity (Kalischuk et al. 2012; Peters et al. 2014; Alkher et al. 2015; Saville and Ristaino 2019). High precipitation and humidity characteristic of BC weather might have contributed to increased late blight occurrence and genotypic diversity. These community and home gardens are often managed by non-expert individuals who obtain their seeds or transplants from diverse locations. In some cases, when infected seeds from previous years are planted, the pathogen overwinters and serves as a new source of inocula in the season. This emphasizes the need for planting clean and disease-free seeds, tubers, and transplants. Moreover, these gardens and farms often act as reservoirs for *P. infestans* populations. These fields are not sprayed with pesticides thus fostering easy pathogen proliferation, and as a result, *P. infestans* populations in these locations might be the most-fit genotypes and endemic in some cases. Therefore, a culmination of these factors including migration of infected plant materials and favorable environmental conditions might have contributed to the increased number of genotypes found in BC and the emergence of novel genotypes recorded in this study.

Although only A1 mating type was found in our study, occurrence of both mating types was reported previously in BC, ON, QC, NB, and PEI (Chycoski and Punja 1996; Kalischuk et al. 2012; Peters et al. 2014). Both mating types were found in close proximity in ON, QC, and PEI in 2011 (Peters et al. 2014). The presence of both mating types in close proximities could increase the chances of sexual recombination resulting in the production of oospores and the emergence of novel *P. infestans* genotypes in BC. These sexual spores can survive extreme weather conditions without the host (Medina and Platt 1999; Mayton et al. 2000). Oospores could allow the pathogen to overwinter without the host and serve as a source of inoculum in subsequent seasons, possibly resulting in the emergence of new recombinant genotypes (Goodwin et al. 1995b; Peters et al. 2014). However, there is little or no direct evidence of oospores in fields to date except for a few instances (Peters et al. 2001) and further epidemiological studies are required from different regions in Canada to understand the existence of oospores in the field. Kalischuk et al. (2012) postulated that sexual recombination contributed to increased genotypic characteristics of *P. infestans* populations in Canada. The emergence of g11 (US 11) might be the result of migration from Mexico (Miller et al. 1997; Peters et al. 2001). Furthermore, the complexity and large genome size of *P. infestans* detailed by whole genome sequencing could lead to higher gene diversity resulting from random genetic drift and mutations (Abu-El Samen et al. 2003; Haas et al. 2009). In addition, *P. infestans* populations can switch from triploid to diploid when exposed to chemical and environmental stresses, such as non-lethal doses of the fungicide metalaxyl and extremely cold winters (Li et al. 2017; Maurice et al. 2019). Therefore, exposure to such stresses could favor sexual reproduction because of the resistant nature of oospores in soil. However, some *P. infestans* populations were reported to self-fertilize (Maurice et al. 2019) and do not require both mating types to produce oospores. This observation indicates that oospores may not only be the result of sexual recombination but also an adaptation to all forms of stress (Li et al. 2017; Maurice et al. 2019).

Overall, the continued dominance of US11 in Pacific western Canada (BC) was found in this study. Our study identified US17 for the first time in Canada and revealed three major existing genotypes (US11, US17, and US8) and 25 new genotypes in BC. Interestingly, the vast majority of the *P*. *infestans* isolates (95%) from BC were resistant to metalaxyl-m. We found the sharp dichotomy in late blight incidence and prevalence between eastern and Pacific western Canada. We also found the continued dominance of US23 in eastern Canada, but this genotype was absent in BC. High genetic diversity was observed among *P. infestans* isolates originating from BC as evidenced by the number of MLGs, genetic diversity indices, and emergence of 25 new genotypes. This suggests that BC is a hotspot for sexual recombination and should be closely monitored for the establishment of a sexual population of *P. infestans*. Also, the emergence of new genotypes and the continued dominance of US11 in BC could pose management challenges. New recombinants may differ in both phenotypic and genotypic characteristics as compared to existing genotypes. Importation of infected plant materials from South America and Mexico was suggested as the potential source of new genotypes into the USA and consequent migration into Canada from the USA (Kalischuk et al. 2012; Peters et al. 2014; Saville and Ristaino 2019). Global movement of plant materials could have contributed to late blight epidemics; hence, there is a need for improved scrutiny of plant materials movement among regions and countries to avoid the migration of genotypes from one region to another. Considering the number of outbreaks each year, the detection of known genotypes such as US11, US17, and US8 and the emergence of novel genotypes from BC as described in this study and other surveys shows that BC is a hub for the establishment of diverse populations. Therefore, close monitoring of *P.* *infestans* populations in this region is useful. Furthermore, more in-depth monitoring of *P. infestans* populations in Canada, especially BC, is required to determine whether sexually reproducing populations are now established, although the recombinants detected in this study were short-lived and not widely spread. Lastly, the need to understand the sources of recombination events and the presence of an ephemeral sexual population as reported by Danies et al. (2014) needs to be studied extensively. In addition to the mechanisms of genetic diversity, gene flow, recombination, and ploidy analysis need to be holistically investigated and genome sequencing of new genotypes and lineages of *P. infestans* will be useful.

## Supplementary Information

Below is the link to the electronic supplementary material.

Supplementary file1 (PDF 139 KB)

## Data Availability

All data generated or analyzed during the study is included in this published article and its supplementary information.
