# Supplementary material for: Genetic structure and population diversity of Phytophthora infestans strains in Pacific western Canada
Source: Appl Microbiol Biotechnol. 2024 Feb 26;108(1):237. doi: 10.1007/s00253-024-13040-6 (PMC10896882; doi:10.1007/s00253-024-13040-6)
Supplement: Supplementary file 1 — Supplementary file1 (PDF 139 KB) [file 253_2024_13040_MOESM1_ESM.pdf]

Applied Microbiology and Biotechnology

Genetic structure and population diversity of *Phytophthora infestans* strains in Pacific western Canada

Segun Babarinde[1,2], Rishi R. Burlakoti[1†], Rick D. Peters[3], Khalil Al-Mughrabi[4], Amy Novinscak[1], Sanjib Sapkota[1], Balakrishnan Prithiviraj[2],

[1]Agassiz Research and Development Centre, Agriculture and Agri-Food Canada, 6947 Hwy 7, Agassiz, BC, V0M 1A0, Canada; [2] Department of Plant, Food and Environmental Sciences, Faculty of Agriculture, Dalhousie University, Truro, NS, B2N 5E3, Canada; [3] Agriculture and Agri-Food Canada, 440 University Avenue, Charlottetown, PE, C1A 4N6, Canada; [4] New Brunswick Department of Agriculture, Aquaculture and Fisheries, 39 Barker Lane, Wicklow, NB, E7L 3S4, Canada

[†]Corresponding author: Rishi R. Burlakoti
email: rishi.burlakoti@agr.gc.ca; Phone:604-316-0501; Fax: 604-796-6133

Supplemental Table S1: Allele size (bp) of different genotypes of *Phytophthora infestans* isolates generated from 12 simple sequence repeat (SSR) loci.

Genotypes †	No. of isolates	Allele size (bp) of SSR markers											
		PiG11	Pi02	PinfSSR11	D13	PinfSSR8	PinfSSR4	Pi04	Pi70	PinfSSR6	Pi63	PinfSSR2	Pi4B
US8	7	156/156	268/268	341/355	108/112	260/266	284/290/294	166/170	192/192	246/246	279/279	173/173	213/225
US11	121	134/156	260/268	331/341	110/110	266/266	284/294/302	160/166/170	192/192	246/246	279/279	173/173	213/213
US17	15	134/156	260/268	331/341	110/110	266/266	284/294	166/170	192/192	246/246	279/279	173/173	213/213
US23 var	5	142/156	268/270	331/341	134/134	260/266	288/294/296	168/170	192/192	246/246	270/279	173/175	213/217
CAC1	1	134/156	270/270	331/341	110/110	266/266	284/294/302	166/170	192/192	246/246	279/279	173/173	213/213
CAC2	2	134/156	260/270	331/341	110/110	266/266	284/294/302	166/170	192/192	246/246	279/279	173/173	213/213
CAC3	1	134/156	260/260	331/341	110/110	266/266	-	166/170	192/192	246/246	279/279	173/173	213/213
CAC4	10	134/156	260/268	331/341	110/110	-	294/302	166/170	192/192	246/246	279/279	173/173	213/213
CAC5	2	134/156	260/268	341/341	110/110	266/266	284/294/302	166/170	192/192	246/246	279/279	173/173	213/213
CAC6	5	134/156	260/268	331/341	110/110	266/266	294/302	160/166/170	192/192	246/246	279/279	173/173	213/213
CAC7	1	134/154	260/268	331/341	110/110	266/266	284/294/302	160/166/170	192/192	246/246	279/279	173/173	213/213
CAC8	1	134/156	260/268	331/341	110/110	260/270	284/294/308	166/170	192/192	246/246	279/279	173/173	213/213
CAC9	1	156/156	260/268	331/341	110/110	266/266	294/302	166/170	192/192	246/246	279/279	173/173	213/213
CAC10	1	156/156	260/268	331/341	110/110	266/266	284/294/302	160/166/170	192/192	246/246	279/279	173/173	213/213
CAC11	2	134/156	260/268	331/341	110/110	266/266	284/294/308	166/170	192/192	246/246	279/279	173/173	213/213
CAC12	1	134/156	260/268	341/355	108/112	260/260	284/290/294	166/170	192/192	246/246	279/279	173/173	213/225
CAC13	10	134/156	260/268	331/341	110/110	266/266	284/294/306	166/170	192/192	246/246	279/279	173/173	213/213
CAC14	4	134/156	260/268	341/341	108/110	266/266	284/294/310	166/170	192/192	246/246	279/279	173/173	213/213
CAC15	1	134/156	260/268	331/341	110/110	266/266	284/294/302	160/170	192/192	246/246	279/279	173/173	213/213
CAC16	2	134/156	260/268 /270	331/341	110/110	266/266	284/294/310	166/170	192/192	246/246	279/279	173/173	213/213
CAC17	1	134/156	260/268	331/341	110/110	266/266	284/294/310	166/170	192/192	246/246	279/279	173/173	213/213
CAC18	1	134/156	260/268	331/341	108/110	266/266	294/310	166/170	192/192	246/246	279/279	173/173	213/213
CAC19	1	134/156	260/268	331/341	110/110	266/266	284/294/306	160/166/170	192/192	246/246	279/279	173/173	213/213
CAC20	1	134/156	260/268 /270	331/341	108/110	266/266	284/294/300	160/166/170	192/192	246/246	279/279	173/173	213/213
CAC21	1	134/156	260/268	331/341	110/110	266/266	284/294/300	160/166/170	192/192	246/246	279/279	173/173	213/213
CAC22	2	134/156	260/268 /270	331/341	110/110	266/266	284/294/300	166/170	192/192	246/246	279/279	173/173	213/213
CAC23	1	134/156	260/268	331/341	110/110	266/266	284/294/300	166/170	192/192	246/246	279/279	173/173	213/213
CAC24	6	134/156	260/268	341/341	108/110	266/266	284/294/302	166/170	192/192	246/246	279/279	173/173	213/213
CAC25	1	134/156	268/268	341/341	108/110	-	284/294	160/166/170	192/192	246/246	279/279	173/173	213/213
CAE1	1	142/156	270/270	331/341	134/134	260/266	288/294	170/170	192/192	246/246	270/279	173/175	213/217

† CAC, Canada coast; CAE, Eastern Canada. Both CAC and CAE genotypes are new (novel) genotypes.
